# Low Microbial Diversity and Abnormal Microbial Succession Is Associated with Necrotizing Enterocolitis in Preterm Infants

**DOI:** 10.3389/fmicb.2017.02243

**Published:** 2017-11-15

**Authors:** Priscila T. Dobbler, Renato S. Procianoy, Volker Mai, Rita C. Silveira, Andréa L. Corso, Bruna S. Rojas, Luiz F. W. Roesch

**Affiliations:** ^1^Centro Interdisciplinar de Pesquisas em Biotecnologia – CIP-Biotec, Campus São Gabriel, Universidade Federal do Pampa, São Gabriel, Brazil; ^2^Serviço de Neonatologia do Hospital de Clínicas de Porto Alegre, Universidade Federal do Rio Grande do Sul, Porto Alegre, Brazil; ^3^Department of Epidemiology, College of Public Health and Health Professions and College of Medicine, Emerging Pathogens Institute, University of Florida, Gainesville, FL, United States

**Keywords:** 16S rRNA gene, metagenomics, preterm morbidities, gut microbiome, ultra-long reads

## Abstract

Despite increased efforts, the diverse etiologies of Necrotizing Enterocolitis (NEC) have remained largely elusive. Clinical predictors of NEC remain ill-defined and currently lack sufficient specificity. The development of a thorough understanding of initial gut microbiota colonization pattern in preterm infants might help to improve early detection or prediction of NEC and its associated morbidities. Here we compared the fecal microbiota successions, microbial diversity, abundance and structure of newborns that developed NEC with preterm controls. A 16S rRNA based microbiota analysis was conducted in a total of 132 fecal samples that included the first stool (meconium) up until the 5th week of life or NEC diagnosis from 40 preterm babies (29 controls and 11 NEC cases). A single phylotype matching closest to the *Enterobacteriaceae* family correlated strongly with NEC. In DNA from the sample with the greatest abundance of this phylotype additional shotgun metagenomic sequencing revealed *Citrobacter koseri* and *Klebsiella pneumoniae* as the dominating taxa. These two taxa might represent suitable microbial biomarker targets for early diagnosis of NEC. In NEC cases, we further detected lower microbial diversity and an abnormal succession of the microbial community before NEC diagnosis. Finally, we also detected a disruption in anaerobic microorganisms in the co-occurrence network of meconium samples from NEC cases. Our data suggest that a strong dominance of *Citrobacter koseri* and/or *Klebsiella pneumoniae*, low diversity, low abundance of *Lactobacillus*, as well as an altered microbial-network structure during the first days of life, correlate with NEC risk in preterm infants. Confirmation of these findings in other hospitals might facilitate the development of a microbiota based screening approach for early detection of NEC.

## Introduction

Necrotizing enterocolitis (NEC) is a major cause of morbidity and mortality in neonatal intensive care units with the majority of cases of NEC occurring among premature infants. The etiology of NEC is complex and appears to be of multifactorial etiology. Prematurity, formula feeding and gut microbiota composition and activities have been proposed as main risk factors for NEC(Niño et al., 2016). Current clinical predictors of NEC remain ill-defined and fairly non-specific. Once clinical signs of NEC are diagnosed, the progression is rapid and associated with high mortality despite aggressive treatment that frequently includes bowel resection (Gregory et al., 2011; Neu and Pammi, 2016). The relative lack of specificity of current diagnostic markers indicates a need for better diagnostic tools to improve early diagnosis of NEC. Due to the advances in Next Generation Sequencing (NGS), identifying alterations in the gut microbiota composition offers a promising approach for advancing our understanding of potential NEC risk factors. Identifying microbial risk signatures could lead to the development of alternative biomarkers for early diagnosis and facilitate novel prevention and treatment strategies. Microbiota sequencing has contributed to establish that initial colonization of the gastrointestinal tract can already occur *in utero* by microbes derived from the maternal microbiota (Jiménez et al., 2008; Mshvildadze et al., 2010; Collado et al., 2016). Other studies suggest that gut microbiota development is affected by the mode of the childbirth (Gritz and Bhandari, 2015) and early feeding type (Jain and Walker, 2015). Earlier studies that focused on identifying single phylotypes that represent pathogens have described a myriad of microbes that correlated with NEC. To date, a specific pathogen that fulfils Koch’s postulates for the etiology of NEC has not been detected (Torrazza and Neu, 2013), and it might not be realistic to demand such stringent criterion. Microbial communities are not a mere collection of individuals but they represent complex and interconnected collection of organisms that communicate, cross-feed, recombine, and co evolve (Layeghifard et al., 2016). Thus, instead of focusing research efforts on detecting individual pathogens associated with development of NEC, the balance of the entire microbial community might be crucial for developing a healthy intestinal microbiota in the neonate that can protect from NEC.

Here, we provide evidence of an association between NEC and distortions in normal microbiota development. Specifically, we detected a decrease in microbial diversity and abundance with age resulting in dominant microbial species associated with NEC cases.

## Materials and Methods

### Experimental Design and Sampling Strategy

This study used a convenience sampling strategy with all patients recruited from the Neonatology Section of Hospital de Clínicas de Porto Alegre (HCPA), Brazil. Pregnant women with gestation age ≤32 weeks that provided written informed consent were enrolled at hospital admission for their delivery. Gestational age was assessed by date of maternal last menstrual period or by ultrasound. The study protocol was approved by the Ethics Committee of Hospital de Clínicas de Porto Alegre (HCPA). Exclusion criteria for mothers were: (1) HIV or congenital infections, (2) recreational drug user or alcohol dependency, and (3) congenital malformations in fetus. Twin pregnancies were not excluded. A total of 568 samples weekly stool samples were collected from diapers of 161 premature infants beginning with the first stool (meconium) until discharge from neonatology. All samples were immediately stored in liquid nitrogen until DNA extraction. Eleven preterm infants were diagnosed with NEC and all their samples were included in the analysis. Twenty-nine control neonates without any NEC symptoms were selected by matching by gestational age, birth mode, antibiotic therapy and birth weight with the NEC cases. The inclusion criteria of the controls were: (1) delivery ≤32 weeks gestational age and (2) no history or symptoms of sepsis. In this study, we analyzed only samples from NEC cases collected up to the 5th week of life as many controls were discharged by that time. The entire dataset was composed of 132 samples.

A neonatologist made the diagnosis of NEC based on the following clinical criteria: abdominal distention, gastric aspirates, bilious vomiting, bloody stools, lethargy, apnea, and hypoperfusion. Pneumatosis intestinalis, indicating air within the subserosal bowel wall, was the radiological hallmark used to confirm the diagnosis in 9 patients. Surgical diagnosis combined with pathology after bowel perforation was used to confirm the diagnosis in the other two patients (Schoeni, 2015).

### Microbial DNA Extraction, 16S Library Preparation, and Sequencing

Microbial DNA was isolated from meconium and fecal samples using the QIAamp Fast DNA Stool Mini Kit (Qiagen, Valencia, CA, United States) following the manufacturer’s instructions. DNA quality was determined by spectrophotometry using NanoVue^TM^ spectrophotometer (GE Healthcare, Chicago, IL, United States). All DNA samples were stored at -80°C until use. The V4 region of the 16S rRNA gene was amplified and sequenced using the PGM Ion Torrent (Thermo Fisher Scientific, Waltham, MA, United States) with the bacterial/archaeal primers 515F and 806R (Caporaso et al., 2012). Multiple samples were PCR-amplified using barcoded primers linked with the Ion adapter “A” sequence (5′-CCATCTCATCCCTGCGTGTCTCCGACTCAG-3′) and Ion adapter “P1” sequence (5′-CCTCTCTATGGGCAGTCGGTGAT-3′) to obtain a sequence of primer composed for A-barcode-806R and P1-515F adapter and primers. Each of the 25 μL of PCR mixture consisted of 2U of Platinum^®^ Taq DNA High Fidelity Polymerase (Invitrogen, Carlsbad, CA, United States), 4 μL 10X High Fidelity PCR Buffer, 2 mM MgSO4, 0.2 mM dNTP’s, 0.1 μM of both the 806R barcoded primer and the 515F primer, 25 μg of Ultrapure BSA (Invitrogen, Carlsbad, CA, United States) and approximately 50 ng of DNA template. PCR conditions used were: 95°C for 5 min, 35 cycles of 94°C per 45 s denaturation; 56°C per 45 s annealing and 72°C per 1 min extension; followed by 72°C per 10 min. The resulting PCR products were purified with the Agencourt^®^ AMPure^®^ XP Reagent (Beckman Coulter, Brea, CA, United States) and the final concentration of the PCR product was quantified by using the Qubit Fluorometer kit (Invitrogen, Carlsbad, CA, United States) following manufacturer’s recommendations. Finally, the reactions were combined in equimolar concentrations to create a mixture composed by 16S gene amplified fragments of each sample. This composite sample was used for library preparation with Ion OneTouch^TM^ 2 System with the Ion PGM^TM^ Template OT2 400 Kit Template (Thermo Fisher Scientific, Waltham, MA, United States). The sequencing was performed using Ion PGM^TM^ Sequencing 400 on Ion PGM^TM^ System using Ion 318^TM^ Chip v2 with a maximum of 40 samples per microchip.

### Metagenomic Sequencing with Ultra-Long Reads

In order to detect the main microbial species associated with NEC cases, Oxford Nanopore MinION^TM^ device was used for sequencing of the gDNA. The same gDNA library used for 16S library preparation and sequencing with Ion Torrent PGM was used for sequencing ultra-long reads. The library was prepared by using the rapid sequencing of genomic DNA for the MinION^TM^ device with the SQK-RA002 kit and the flow cell FLO-MIN 107 R9 version following the manufacturer’s instructions. The raw reads were acquired through the MinKNOW software in a 22.5 h run experiment; the data analysis was performed with What’s in My Pot (WIMP) workflow for sequence classification.

### 16S Sequence Processing for Downstream Analyses

The FastQ files exported from the Ion PGM^TM^ System were analyzed following the recommendations of the Brazilian Microbiome Project (Pylro et al., 2014) using the BMP Operating System (BMPOS) (Pylro et al., 2016). Briefly, the OTU (Operational Taxonomic Unit) table was built using the UPARSE pipeline (Edgar, 2013) in which the reads were truncated at 200 bp and quality filtered using a maximum expected error of 0.5. Filtered reads were de-replicated and singletons were removed. The sequences were clustered into OTUs at 97% similarity cut-off, checked for chimeras, and representative sequences were obtained for each microbial phylotype (Edgar, 2013). Taxonomic classification was carried out in QIIME (Caporaso et al., 2010) based on the UCLUST method against the Greengenes 13.5 database (McDonald et al., 2012) with a confidence threshold of 80%. Sampling effort was estimated using Good’s coverage (Good, 1953).

### Statistical Analysis

Clinical data was imported into the R environment (R Development Core Team, 2008). The normality of data was tested by Shapiro–Wilk *W* test (*p* > 0.05).

Quantitative variables with normal distribution were described through means/SD and compared by the Student’s *t*-test. Quantitative variables with non-normal distribution were described through median/interquartile range and compared by the Mann–Whitney test. Qualitative variables were described by frequencies/percentiles and compared Fisher’s Exact Test.

All 16S rRNA gene libraries were normalized by randomly resampling the sequence data to the same number of sequences found in the smallest library according to the recommendations of Lemos et al. (2011). The BIOM file was imported into the R environment (R Development Core Team, 2008) and a compositional dissimilarity matrix was generated based on Euclidean distances between samples using the “phyloseq” package (McMurdie and Holmes, 2013). The matrix was used in a non-parametric permutational Multivariate Analysis of Variance (perMANOVA) with the *Adonis* function available in the vegan package (Oksanen et al., 2015) for detecting confounding variables. Calculations of microbial dominance and the Shannon diversity index were obtained and plotted using the “phyloseq” package (McMurdie and Holmes, 2013). Differences in alpha diversity measurements and phyla abundance were tested on weekly samples.

A co-occurrence network analysis was performed at the microbial species level. To test for co-occurrence patterns, poorly represented OTUs (OTUs with less than five sequences) were removed and species that were present in at least 70% of the samples were identified and discarded (species present in less than 70% of all samples of each group were excluded) generating one dataset per patients group. For network inference, the SparCC (Friedman and Alm, 2012) procedure was adopted. Co-occurrence was considered robust when the correlations (either positive or negative) were both ≥0.7 and statistically significant (*p*-value ≤ 0.05). The network was explored and visualized with the interactive platform gephi (Bastian et al., 2009).

Biomarker identification and classification was performed by Random Forests (Breiman, 2001) through the randomForest package implemented in the R environment (R Development Core Team, 2008). The analysis was performed using only meconium samples, at OTU level (97% similarity cutoff) using 5000 trees and a minimum library size of 1070 sequences rarefied by subsample without replacement. Only dominant OTUs with an abundance greater than 1% in each individual sample were kept. In an attempt to remove any possible noise caused by antibiotic usage, the differential abundance test was performed using DESeq2 method implemented in Qiime (Caporaso et al., 2010) with the same dataset of meconium samples using antibiotic usage as variable. OTUs with differential abundance caused by antibiotic usage were excluded from the biomarker identification analysis.

## Results

### Subjects Characteristics

A total of 132 samples from the first stool (meconium) until the 5th week of life were obtained from 40 preterm infants (29 controls and 11 NEC cases) selected in this study. The characteristics of the preterm infants are shown in **Table 1**. None of the metadata tested, including known confounding variables for NEC and microbial colonization (e.g., gestational age, birth weight, birth mode, and antibiotics usage), was significantly different between the control and NEC groups. In term of feeding regimen, in the NEC group three subjects did not receive any enteral feeding before NEC diagnosis, one received only mothers’ breast milk, one received only formula and six received mixed feeding (breast milk and formula). In the control group, two patients received only breast milk and the other 27 received mixed feeding (breast milk and formula). Seven patients (63.6%) in the NEC group received a short course of antibiotic (3 days) before NEC, and 18 patients (62%) in the control group received a short course of antibiotic (3 days) in the first 2 weeks of life. No patient in either groups had a positive blood culture. Causes of preterm delivery were: 6 (54.5%) of the NEC group and 12 (41.3%) of the control group due to spontaneous preterm labor, 5 (45.5%) and 11 (37.9%) of the NEC group and controls, respectively, were induced due to maternal preeclampsia. Other causes of premature births were placental rupture (10.3% in controls) and intrauterine restriction growth (7% control group). In two patients in the NEC group the diagnosis was based on surgical inspection and by anatomopathological exam; they showed no pneumatosis intestinalis on radiographies but had bowel perforation requiring surgery and confirmation of NEC. Ten (91%) of the NEC group and 28 (96.5%) controls, respectively, required ventilatory support.

**Table 1:** Characteristics of the studied newborns.

	NEC (*n* = 11)	Controls (*n* = 29)	*p*-value
Gestational age (weeks)	29.75 (±2.16)	31.14 (±1.61)	0.072^*^
Rupture of membranes (hours)	0 (0-28)	0 (0-6.5)	0.976^*^*
Vaginal birth	4 (36.36%)	5 (17.24%)	0.227ф
Intrapartum antibiotic therapy	5 (45.45%)	15 (51.72%)	0.999ф
Birth weight (g)	1235 (±411.12)	1529 (±474.40)	0.078^*^
Age at NEC diagnosis (days of life)	8.0 (5.0-13.0)	–	–
Pneumatosis intestinalis	9 (81.82%)	–	–
Histologic chorioamnionitis	4 (36.4%)	8 (27.6%)	0.704ф
Maternal diabetes	0 (0%)	6 (20.7%)	0.162ф
Pregnancy preeclampsia	5 (45.5%)	11 (37.9%)	0.728ф

*Variables were described as mean/SD, median/quartiles, and frequency/percentile. ^∗^*t*-test; ^∗∗^Mann–Whitney *U* Test*; ^ϕ^*Fisher’s Exact Test.*

### Overall 16S Sequencing Report and Sequencing Coverage

After quality filtering the 16S rRNA reads, a total of 717,201 high-quality sequences longer than 200 bp were retained. To analyze how well sequences from each sample were representative of the underlying bacterial community, sequence coverage was calculated (Supplementary Table S1). Before rarefying to the same number of sequences/sample coverage at the 97% similarity level for OTU ranged from 95.8 to 100%. After rarefaction, coverage ranged from 89 to 100% indicating a sufficient number of 16S sequences for a representative microbiota analysis.

### Early Identification of Signature Taxa Correlated with Disease Risk

To identify signature bacteria associated with the risk for NEC development, we performed a biomarker screening analysis using Random Forests, which uses multiple learning algorithms to generate classifications based on decision trees. As we were interested in early detection of biomarkers differentiating NEC *versus* Controls Random Forest analyses were performed only with meconium samples.

It’s important to note that our analysis for detecting signature bacteria as risk markers for NEC development might have been influenced by antibiotic usage. In an attempt to remove this effect, we performed a differential abundance analysis comparing microbial communities with and without antibiotic usage (Supplementary Table S2). Three OTUs belonging to the *Prevotella* and *Pseudomonas* genera and to the species *Streptococcus anginosus* were strongly affected by antibiotics use. They were further classified and consequently removed from our dataset.

By using Random Forests, we were able to identify signature OTUs discriminating controls and NEC cases in meconium samples (collected before NEC diagnosis). The 10 most significant bacterial OTUs for classifying meconium samples into NEC or Control are presented in **Table 2**. The estimate of error rate (Out-of-Bag, OOB) of this analysis was 24% meaning we could predict the microbial biomarkers for disease with 76% accuracy. For each OTU we provide a mean decrease accuracy (MDA) value that could be interpreted as the loss of accuracy in the classification of the model when a specific OTU is removed. Therefore, OTUs with a large mean decrease in accuracy values were more important for classification of that OTU as a microbial biomarker. The OTU with the greatest mean decrease accuracy was identified as belonging to the *Enterobacteriaceae* family (MDA = 6.9%) and was associated with the NEC group. Other OTUs identified as belonging to the genera *Butyricimonas* and *Bacteroides* and the family *Microbacteriaceae* were associated with NEC and presented a MDA of 4.9, 4.7, and 4.1%, respectively.

**Table 2:** The most important bacterial OTUs for predicting microbial biomarkers for early detection of Necrotizing Enterocolitis from meconium samples according to the Random Forests analysis.

Closest microbial relative						Mean decrease accuracy	OUT responsible for differentiate between	
Phylum	Class	Order	Family	Genus	Species		NEC	Control
*Proteobacteria*	*Gammaproteobacteria*	*Enterobacteriales*	*Enterobacteriaceae*	*–*	*–*	6.91	Yes	No
*Firmicutes*	*Bacilli*	*Lactobacillales*	*Lactobacillaceae*	*Lactobacillus*	*–*	6.90	No	Yes
*Bacteroidetes*	*Bacteroidia*	*Bacteroidales*	*Bacteroidaceae*	*Bacteroides*	*–*	5.84	No	Yes
*Firmicutes*	*Bacilli*	*Lactobacillales*	*Lactobacillaceae*	*Lactobacillus*	*iners*	5.70	No	Yes
*Bacteroidetes*	*Bacteroidia*	*Bacteroidales*	*[Odoribacteraceae]*	*Butyricimonas*	*–*	4.96	Yes	No
*Bacteroidetes*	*Bacteroidia*	*Bacteroidales*	*Bacteroidaceae*	*Bacteroides*	*–*	4.69	Yes	No
*Proteobacteria*	*Gammaproteobacteria*	*Pseudomonadales*	*Pseudomonadaceae*	*Pseudomonas*	*–*	4.46	No	Yes
*Proteobacteria*	*Alphaproteobacteria*	*Rhizobiales*	*Bradyrhizobiaceae*	*–*	*–*	4.37	No	Yes
*Actinobacteria*	*Actinobacteria*	*Actinomycetales*	*Microbacteriaceae*	*–*	*–*	4.15	Yes	No

Moreover, OTUs identified as *Lactobacillus* sp. and *Lactobacillus iners* were highly associated with controls (**Table 2**). Multiple other OTUs were also associated with controls; at the family level *Bradirhizobiaceae* and at the genus level *Bacteroides*. The lack or low abundance of these OTUs in NEC cases indicates that not only might pathogenic bacteria be enriched in NEC cases but beneficial bacteria that confer protection might be lacking.

### Identification of Microbial Species Previously Detected As Signature Taxa Correlated with Disease Risk

After an OTU identified as *Enterobacteriaceae* was recognized to strongly correlate with NEC, a metagenome analysis with ultra-long sequences was performed on the NEC sample that had the greatest abundance of this OTU. This approach removed potential PCR bias and allowed for microbial identification based on metagenome sequences, extending our findings from the 16S based analysis.

**FIGURE 1:**
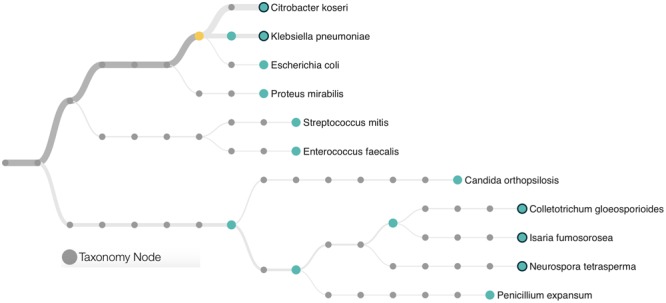
Phylogenetic classification scheme of the microbial community found in a patient with further development of Necrotising Enterocolitis (NEC). Species identification was based in ultra-long metagenome sequences matching the NCBI taxonomy. The tree presents only microorganisms with a minimum of 10 classifications per node and a threshold quality score of 20. Branches are weighted according to the microbial abundance. The yellow circle represents the node of the *Enterobacteriaceae* family.

A total of 22,333 sequences with a mean length of 2,398 bases and a maximum read length of 735,426 bases were obtained. Those sequences were analyzed using the “What’s in My Pot” (WIMP) workflow which is designed to detect organisms from a metagenome library for which a reference is available via the NCBI RefSeq database for Viruses, Bacteria, Fungi, and Archaea. A tree with the detected species based on NCBI taxonomy is presented in **Figure 1** and the entire report is presented in the **Supplementary Figure S1**. The WIMP workflow places the reads at the species or sub-species level. If there is insufficient information for species level identification, the read is placed at a higher rank of the taxonomic tree. If no placement is sufficiently reliable, the sequence is labeled as “Unclassifed”. A total of 8,844 sequences were classified and 13,489 remained unclassified (without any mach in the NCBI RefSeq database). We detected three species of the *Enterobacteriaceae*: *Citrobacter koseri* (average quality score of 24), *Klebsiella pneumoniae* (average quality score of 23), and *Escherichia coli* (average quality score of 23). A *q*-score greater than 20 represents a probability greater than 99% for the match, suggesting that all three matches can be made with high confidence. The *Citrobacter koseri* was the most abundant microbial species comprising 15% of the total number of classified sequences followed by *Klebsiella pneumoniae* with 3% *Escherichia coli* with 0.16%.

This metagenome sequencing also allowed us to detect six distinct species of Eukaryotes from the *Saccharomyceta* class. They were *Penicillium expansum, Isaria fumosorosea, Metarhizium acridum, Colletotrichum gloeosporioides, Neurospora tetrasperma*, and *Candida orthopsilosis.* Altogether, those fungi made up 0.38% of the total microbial community. No virus or Archaea was detected within this dataset.

### Microbial–Microbial Competition Create a Healthy Gut Environment

**FIGURE 2:**
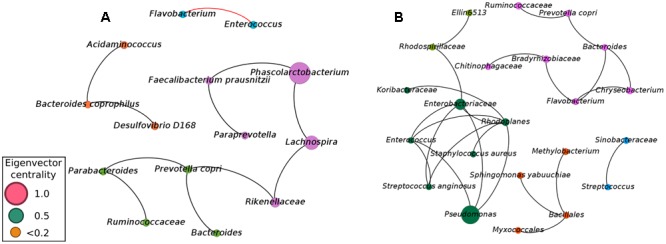
Co-occurrence network of the microbial community found in meconium samples (first evacuation) of preterm infants not diagnosed with NEC **(A)** and preterm infants with subsequent diagnostic of NEC **(B)**. A connection stands for a strong Pearson’s correlation (*p* ≥ 0.7 and *p*-value ≤ 0.05). Each node represents an Operational Taxonomic Unity (OTU) grouped at 97% similarity cutoff and node sizes are proportional to the value of eigenvector centrality of each taxa. Black lines represent the positive significant correlations and red lines represent the negative significant correlations. The colors of the circles represent the bacterial modules.

Although our approach was capable of providing sufficient evidence for detecting microbial biomarkers for early diagnosis of NEC, we also focused efforts on analyzing the balance of the entire microbial community. Considering microbial communities as a complex and interconnected collection of organisms that communicate, cross-feed, recombine, and co- evolve, a microbial network analysis was applied in order to obtain a better view of the microbial–microbial competition in cases of NEC and healthy controls. Network analysis (using meconium samples) was applied to identify microbial correlations essential for community assembly and/or stability. The co-occurrence networks from the controls showed a total of 14 nodes (phylotypes) and 11 edges (connections between phylotypes), with 10 positive and 1 negative correlation (**Figure 2**). The network of NEC cases had 22 nodes and 24 edges, with only positive correlations. The network of controls had an average clustering coefficient of 0 (the extent to which nodes are embedded in their neighborhood, and therefore, the probability that two co-occurring taxa also co-occur with a third taxa), an average path length of 1.81 (the average network distance between all pairs of nodes) and modularity of 0.599 (values >0.4 suggest that the network has modular structure). The network of NEC cases presented an average clustering coefficient of 0.121, an average path length of 1.324 and modularity of 0.622. These observations indicate that the co-occurrence network from infants that will develop NEC is slightly more clustered than in the controls, indicating higher interspecies correlations. The taxa present in the network of NEC cases were more modular, with communities more densely related, than in the controls.

To determine how influential individual microbial phylotypes were, we calculated the eigenvector centrality for each node in the network. *Phascolarctobacterium* was the most influential phylotype in controls while *Pseudomonas* was the most influential in the network of NEC cases. *Pseudomonas* and *Enterobacteriaceae* were entirely absent in controls, while in the NEC cases *Pseudomonas* was positively associated with *Enterobacteriaceae* and belonged to a dominant community. This community comprised the ‘core’ network of infants that later developed NEC and included potential pathogens such as *Pseudomonas, Enterococcus, Enterobacteriaceae*, and *Staphylococcus aureus* (**Figure 2B**). Importantly, the OTU classified as *Enterobacteriaceae* was the same one previously detected by Random Forests analysis as a strong microbial biomarker for early detection of NEC.

In the network from the controls, *Enterococcus* was negatively associated with *Flavobacterium*, which could play a role in the maintenance of a healthy gut environment (**Figure 2A**). Moreover, the three most influential phylotypes in the control network were assembled into a microbial cluster comprised of only the obligatory anaerobic *Phascolarctobacterium, Faecalibacterium prausnitzii, Paraprevotella, Rikenellaceae*, and *Lachnospira* (**Figure 2A**). This community was present in the gut microbiota of controls while entirely absent in infants with future diagnosis of NEC.

### Low Microbial Diversity and Chaotic Microbial Succession in NEC Cases

**FIGURE 3:**
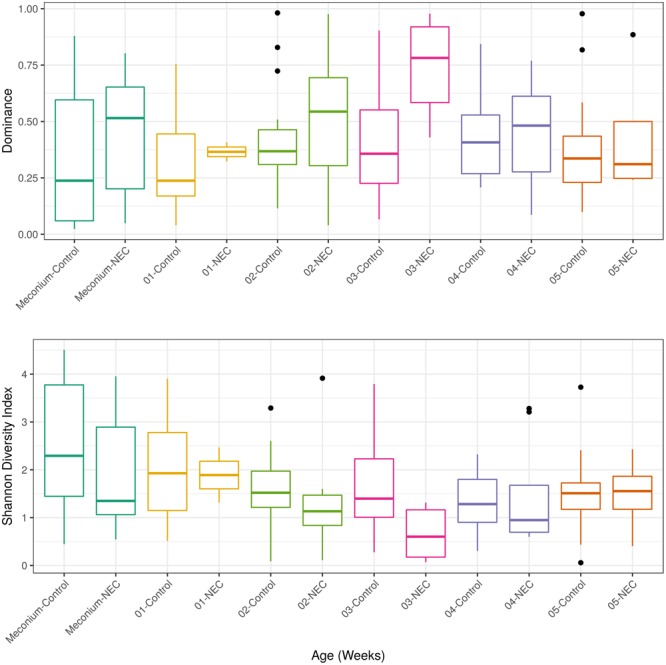
Alpha diversity measurements of microbial communities in meconium and fecal samples from preterms diagnosed with NEC and preterms used as control without NEC diagnosis beginning with the first stool (meconium) and continuing until the 5th week of life. The upper panel presents the Dominance of microbial phylotypes. The bottom panel presents the Shannon microbial index of diversity. Boxes span the first to third quartiles; the horizontal line inside the boxes represents the median. Whiskers extending vertically from the boxes indicate variability outside the upper and lower quartiles, and the black circles indicate outliers. Samples are colored by age: Meconium (first pass stool between 0 and 4 days of life), first week (5–7 days) until the 5th week.

After detecting a microbial biomarker associated with NEC and microbial correlations essential for community assembly and/or stability of a healthy community in meconium samples the next step was to analyze longitudinal microbial community changes up to the 5th week of life. Microbial diversity analysis was performed for evaluating differences between NEC cases and controls at each week of sampling (**Figure 3**). Patients with NEC tended to exhibit low microbial diversity and high dominance compared to the controls even though significant differences (at level 0.05) between treatments were observed only during the 3rd week (**Figure 3**). During week 3, microbial diversity was greater in controls (*p*-value = 0.01), with a mean Shannon diversity index of 1.58 in controls and 0.66 in NEC cases. Microbial dominance at the 3rd week was 0.74 in NEC cases and 0.41 in the controls *(p-*value = 0.01).

**FIGURE 4:**
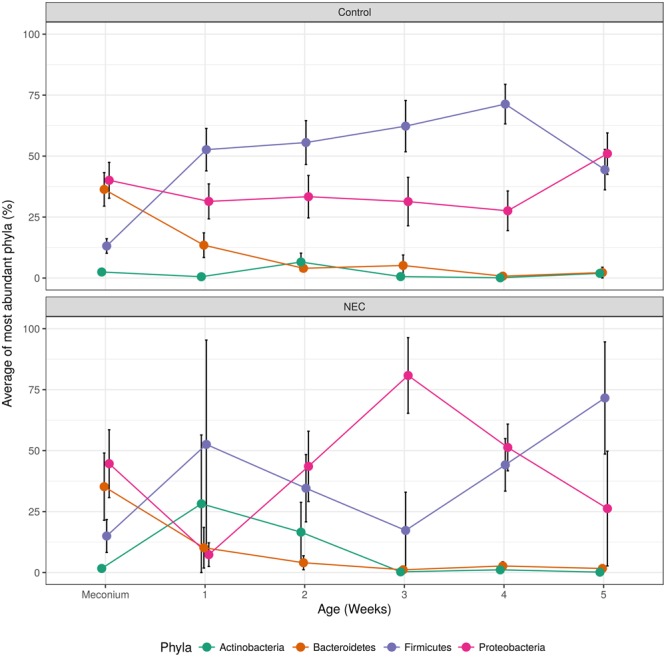
Over time changes of most abundant microbial phyla found in controls **(top)** and NEC **(bottom)**. The black vertical bars indicate the standard error of the average.

After important changes in microbial diversity were detected during the infant’s’ development we evaluated the shifts of the main microbial phyla over time. Infants who developed NEC presented an indeterminate pattern of microbial succession (called here ‘chaotic’ or ‘abnormal’ pattern) when compared to the controls (**Figure 4**). The gut microbial development of infants without NEC was characterized by a shift in the most dominant microbial phyla between the period of 0–4 days and the period of 5–7 days (first week) of life. In the first 4 days of life, the gut microbiota of controls was marked by high abundance of *Proteobacteria* (average of 40.07%) and *Bacteroidetes* (36.35%), and low abundance of *Firmicutes* (13.14%) and *Actinobacteria* (2.47%). During days 5–7 of life, the gut microbiota of controls was dominated by *Firmicutes* (52.64%) and *Proteobacteria* (31.43%) with low abundance of *Bacteroidetes* (13.47%) and *Actinobacteria* (0.54%). Similarly, in the first four days of life the gut microbiota of the NEC group was marked by dominance of *Proteobacteria* (44.66%) and lower abundance of *Bacteroidetes* (35.23%), *Firmicutes* (15%), and *Actinobacteria* (1.7%). However, contrary to the subtle decrease of *Proteobacteria* in the controls during week 1, the decrease of *Proteobacteria* (*p*-value = 0.026) in NEC group was abrupt, followed by a bloom of *Proteobacteria* from week 2 to 3 and then a steady decline through weeks 4 and 5 that coincided with an increase in *Firmicutes*. In contrast, in controls we observed a steady increase in *Firmicutes* overlapping with a decline in *Proteobacteria, Bacteroidetes*, and *Actinobacteria* until week 4 followed by a sudden re-emergence of *Proteobacteria*. During week 3 the higher abundance of *Proteobacteria* (*p*-value = 0.024) and lower abundance of *Firmicutes* (*p*-value = 0.039) in the NEC group reached statistical significance. In addition, in NEC cases we observed an overall higher average abundance of *Actinobacteria* than in controls during weeks one and two (28.21 and 16.57%, respectively).

### Controlling for Confounding Variables

For controlling possible confounding variables, the samples were stratified into meconium and feces and a Permutational Multivariate Analysis of Variance was performed (**Table 3**). The analysis of overall microbial community structure revealed strong differences between meconium and stool samples (*p*-value = 0.001) (**Table 3**). Although the difference was significant the percentage of variation in distances explained by type of sample (meconium or feces) was only 7.1% (*R*^2^ = 0.071). The meconium microbial community structure showed no correlation with antibiotic usage by the infants, weight at birth, mother’s antibiotic usage prior to delivery, delivery mode (C-section or vaginal birth) and gestational age. Fecal microbial community composition at later time points did correlate with type of birth, however, only with the low value of *R*^2^ (3%) Antibiotic usage, by either the newborn or mother, birth weight or gestational age did not influence the microbial community structure of the fecal samples (**Table 3**).

**Table 3:** Results of perMANOVA analysis of the Euclidean dissimilarities for bacterial OTU community structure used for controlling confounding variables.

	*DF*	*SS*	*F*	*R*^2^	*p-*value
**All samples**					
Sample (meconium or feces)	1	3936380	10.049	0.07176	**0.001**
Residuals	130	50921285		0.92824	
Total	131	54857665		1.00000	
**Meconium samples**					
Antibiotic (infant)	1	309360	1.02914	0.03188	0.352
Weight at birth	1	253581	0.84358	0.02614	0.448
Antibiotic (mother)	1	91317	0.30378	0.00941	0.903
Delivery mode (C-section/vaginal)	1	691139	2.29918	0.07123	0.093
Gestational age	1	237954	0.79159	0.02452	0.485
Residuals	7	2104215		0.21687	
Total	32	9702507		1	
**Fecal samples**					
Antibiotic (infant)	1	561334	1.4862	0.01361	0.131
Weight at birth	1	437570	1.1585	0.01061	0.275
Antibiotic (mother)	1	628613	1.6643	0.01524	0.108
Delivery mode (C-section/vaginal)	1	1428960	3.7833	0.03464	**0.001**
Gestational age	1	421011	1.1147	0.01021	0.291
Residuals	62	23417339		0.56764	
Total	98	41253964		1	

*Df, degrees of freedom; SS, sum of squares; *F, F*-value by permutation. Bold indicates statistical significance (*p*-value < 0.05); *p*-values are based on 999 permutations.*

## Discussion

Technological advances in our ability to analyze complex microbial communities and to detect minute amounts of potentially pathogenic microbial agents have opened new avenues for investigating microbial contributions to preterm disease risk (Mshvildadze et al., 2010). In this study, we characterized the intestinal microbiota of Brazilian preterm infants with NEC development and controls in fecal samples collected from birth until the 5th week of life. We minimized variation by matching cases with controls based on clinical variables that can affect microbiota composition.

Gestational age and birth weight have been discusses as crucial sources of microbiota variation (Penders et al., 2006; Hill et al., 2017), In our study all infants were preterm and the difference between groups was only 1-week gestational age and 273 g birth weight; unlikely sufficient to invalidate our conclusions.

Another confounding variable is the birth mode (C-section/vaginal). (Bokulich et al., 2016; Yassour et al., 2016; Stewart et al., 2017). While the role of birth mode apparently remains a controversial question, in our dataset, 36 and 17% of the infants were vaginally born in NEC cases and controls, respectively. The birth mode significantly affected the gut microbial structure (as judged by the *p-*value shown in **Table 3**) in fecal samples, but not in meconium. This is expected because gastrointestinal tract colonization can already occur *in utero* by microbes derived from the maternal microbiota (Jiménez et al., 2008; Mshvildadze et al., 2010; Collado et al., 2016) not being affected by delivery mode.

Antibiotic administration is one of the most important community disturbances of the human microbiota (Zhao et al., 2015) with pronounced short and long term effects (Jernberg et al., 2007; Panda et al., 2014; Rashid et al., 2015; Isaac et al., 2017). Ideally, for prospective studies like ours, the use of antibiotics should be either a stratification factor or an exclusion criteria. However, it is rare to find enough patients who have not undergone antibiotic treatment to sufficiently power the study nor is it ethical to take a patient off a drug that is controlling a disease for the purpose of a study (Devkota, 2016). Within the samples analyzed here, 63% infants in the NEC group received antibiotics previous to NEC diagnosis and 62% patients in the control group received antibiotic in the first 2 weeks of life. As we could not exclude those infants due to power concerns, we instead detected the main microbial OTUs affected by antibiotic therapy using differential abundance analysis (see Supplementary Table S2) and excluded affected taxa from the analysis.

Another important factor for shaping the babies microbiota is diet. Type of feeding may interfere greatly in the regulation of the intestinal microbiota. Brest-fed infants present a fecal microbiota enriched by *Bifidobacterium* (Fallani et al., 2010; Bezirtzoglou et al., 2011). In our study, all meconium samples were obtained before feeding; thus, diet should not affect our detection of microbial biomarkers.

Differences in first evacuation (meconium) are difficult to detect due to the high variability between newborns and the very low abundance of many species (Hu et al., 2013; Ardissone et al., 2014). Nevertheless, we identified an altered gut microbiota in infants prior to the onset of NEC. In our longitudinal analysis, we observed a chaotic pattern of community changes in NEC cases, whereas in controls we observed a steady increase in *Firmicutes* coinciding with a decline in *Proteobacteria, Bacteroidetes*, and *Actinobacteria* until the 4th week of life. Contrary to the subtle decrease in *Proteobacteria* in controls, NEC cases presented with a bloom of *Proteobacteria* from week 2 to 3.

Recently, Pammi et al. (2017) performed a systematic review and meta-analyses of stool microbiome profiles in preterm infants to distinguish and describe microbial dysbiosis prior to the onset of NEC. The review included 14 studies (since 1996 upto 2016) and nine of them provided sequences and clinical data to perform the meta-analyses. This review revealed increased relative abundances of *Proteobacteria* and decreased relative abundances of *Firmicutes* and *Bacteroidetes* prior to the onset of NEC. These results are in line with our findings, however, all studies investigated by Pammi et al. (2017) were conducted in North America and Europe. We are confirming this microbial pattern prior to NEC onset for the first time in South America. This is important to note because a few studies point to differences in gut microbiome across locations (Yatsunenko et al., 2012) and even between different neonatal care unities in close proximity (Brooks et al., 2014; Hartz et al., 2015; Ohoka and Ito, 2016).

A loss of bacterial diversity in the gut has previously been linked to poor outcomes in preterm infants, as well as to other disorders such as late onset sepsis (Madan et al., 2012). During the third week of age, the gut microbiota of cases was dominated by either *Pseudomonas* or *Enterobacteriaceae*, both belonging to the *Gammaproteobacteria* class, while microbiota in controls was dominated by bacteria from the phylum *Firmicutes*. Besides ischemic injury, Dicken et al. suggest that bacterial overgrowth and colonization of the small intestine with Gram-negative bacteria are important factors associated with bowel dysmotility in NEC Dicken et al. (2011).

Recent microbiome studies suggest various health implications of distorted commensal microbiota composition in preterm infants. A prospective preterm infant study in the United States (Mai et al., 2011) reported a 34% increase of *Proteobacteria* and a 32% decrease in *Firmicutes* in NEC cases between 1 week and <72 h before diagnosis. They suggested that a microbial signature characterized by increased *Enterobacteriaceae* identified in NEC cases, contributed to disease etiology. A study in Europe (Heida et al., 2016) detected *Enterobacteriaceae* in 50% of the 16S rRNA bacterial sequences in newborn’s feces samples but that did not correlate with NEC. In that study the presence of *Clostridium perfrigens* was positively associated with NEC even in early meconium samples. Overall, *Enterobacteriaceae* appear to be the most commonly described bacteria correlating with NEC; *Clostridium* sp. and *Staphylococcus* sp. follow (Wang et al., 2009; Strunk et al., 2011; Stewart et al., 2012). A single pathogenic species might not represent the entire etiology of NEC as the underlying microbiota community might contribute to disease risk. Indeed, multiple enteric pathogens that are strongly associated with diarrhoea in US children were detected in healthy children from developing countries, suggesting that either underlying resistance or microbiota composition might provide protection (Pop et al., 2014).

Our results are consistent with others that suggest *Enterobacteriaceae* family as the main microbial contributor to NEC risk. Shotgun sequencing further confirmed our initial 16S rDNA based observation. Despite obtaining high quality reads, many shot gun sequences fail to be classified at the genus or species levels due to their short length. This pitfall was overcome here by shot gun sequencing ultra-long reads with the 3rd generation sequencer Nanopore MinION^TM^. With this approach, we detected two microbial species from the *Enterobacteriaceae* family, *Citrobacter koseri* and *Klebsiella pneumoniae*, that might represent potential microbial biomarkers for early diagnosis of NEC.

Our metataxonomic approach allowed us to go beyond detection of the presence of potential key microbes involved in NEC pathogenesis by identifying alterations in gut microbial community including the correlations among different microbial species using co-occurrence network analysis. Preterms were shown previously to have an impaired early colonization of obligate anaerobes when compared to healthy full term newborns and subsequent transition to a more anaerobic environment (Arboleya et al., 2012). In our co-occurrence network analysis, we found that a community of obligate anaerobes was highly influential in the intestine of controls during the first four days of life and appeared to control the proliferation of *Enterobacteriaceae*. This protective population was absent in the NEC cases. *Faecalibacterium prausnitzii* was included in this cluster, and it was previously suggested to have a protective role in the gut mucosa through tightening the gut barrier (Martín et al., 2015).

## Conclusion

Decreased diversity and differential abundance of *Enterobacteriaceae*, as well as an altered community structure, during the first four days of life, correlated with increased risk for developing NEC. The NEC infants’ gut microbiome appeared chaotic with unstable succession of dominant phyla; while healthy premature control infants’ microbiomes lacked abrupt disruptions, and reached a more balanced composition by the fifth week of life. Our data suggests that early detection of (i) high dominance of *Enterobacteriaceae*, especially *Citrobacter koseri* and *Klebsiella pneumoniae*, (ii) lack of *Lactobacillus*, (iii) low diversity, and (iv) altered microbial–microbial associations during the first days of life could be indicators of NEC risk in preterm infants in a Brazilian NICU. Once confirmed at other locations our findings can inform the development of risk biomarkers that can facilitate improved early diagnosis of NEC.

## Data Availability

The datasets generated during the current study are available in the NCBI repository with the following accession number SRX2546896/Metagenome of preterm infants with Necrotizing Enterocolitis.

## Ethics Statement

The study protocol was approved by the Ethics Committee of Hospital de Clínicas de Porto Alegre (HCPA). Pregnant women with gestation age ≤32 weeks that provided written informed consent were enrolled at hospital admission for their delivery.

## Author Contributions

LR and VM conceived the project and the experimental designs. LR and PD performed the experiments, interpreted the results, generated the figures, and wrote the manuscript. VM provided guidance for gut microbiota analysis and interpreted the results and wrote the manuscript. RP, AC, BR, and RS collected the samples and the metadata and performed the analysis of the clinical data.

## Conflict of Interest Statement

The authors declare that the research was conducted in the absence of any commercial or financial relationships that could be construed as a potential conflict of interest. The reviewer ES-T and handling Editor declared their shared affiliation.

## Supplementary Material

FIGURE S1 WIMP report of the ultra-long metagenomic reads analysis using Nanopore MinION^TM^ device.
