# Supplementary figures and images for: Low Microbial Diversity and Abnormal Microbial Succession Is Associated with Necrotizing Enterocolitis in Preterm Infants
Source: Front Microbiol. 2017 Nov 15;8:2243. doi: 10.3389/fmicb.2017.02243 (PMC5695202; doi:10.3389/fmicb.2017.02243)

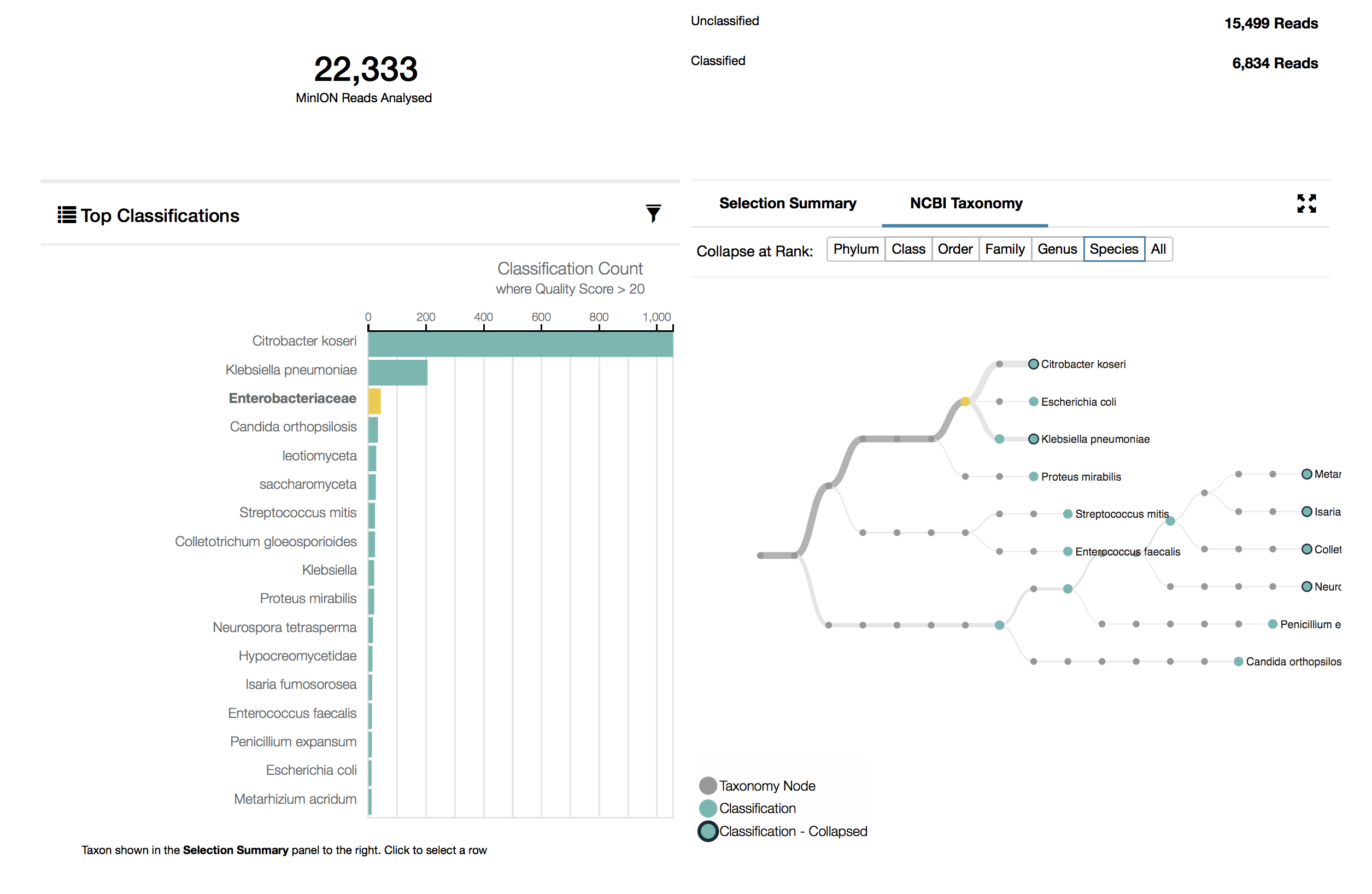

Supplement: FIGURE S1 — WIMP report of the ultra-long metagenomic reads analysis using Nanopore MinIONTM device. [file Image_1.TIFF]
