# Supplementary material for: Low Microbial Diversity and Abnormal Microbial Succession Is Associated with Necrotizing Enterocolitis in Preterm Infants
Source: Front Microbiol. 2017 Nov 15;8:2243. doi: 10.3389/fmicb.2017.02243 (PMC5695202; doi:10.3389/fmicb.2017.02243)
Supplement: Supplementary file 2 [file Table_1.DOCX]

**Table S1.** Good’s coverage and number of OTUs at 97% similarity cutoff of all samples used in the analysis.

| SampleID | Good’s Coverage Pre-Rarefaction | Good’s Coverage Post-Rarefaction | Number of Distinct OTUs Pre-Rarefaction | Number of Distinct OTUs Post-Rarefaction |
| --- | --- | --- | --- | --- |
| P10R2 | 0.989950 | 0.96276 | 121 | 65 |
| P10R3 | 0.999559 | 0.99814 | 23 | 13 |
| P11R1 | 0.982724 | 0.88827 | 416 | 188 |
| P11R2 | 0.978076 | 0.96741 | 103 | 67 |
| P11R3 | 0.999436 | 0.99348 | 22 | 12 |
| P11R4 | 0.999556 | 0.99814 | 26 | 15 |
| P11R5 | 0.99928 | 0.99534 | 38 | 20 |
| P13R1 | 0.991405 | 0.93762 | 330 | 115 |
| P13R2 | 0.989884 | 0.97858 | 63 | 36 |
| P13R3 | 0.999454 | 0.99628 | 25 | 10 |
| P13R4 | 0.999255 | 1 | 9 | 8 |
| P14R1 | 0.994094 | 0.92644 | 297 | 134 |
| P14R2 | 0.991441 | 0.99069 | 19 | 17 |
| P14R4 | 0.998631 | 0.99534 | 14 | 11 |
| P14R5 | 0.99775 | 0.99441 | 28 | 20 |
| P15R1 | 0.996015 | 0.96741 | 173 | 122 |
| P16R1 | 0.996067 | 0.9702 | 163 | 116 |
| P16R2 | 0.988974 | 0.96927 | 81 | 53 |
| P16R3 | 0.994591 | 0.99069 | 15 | 14 |
| P16R4 | 0.997395 | 0.99814 | 21 | 14 |
| P16R5 | 0.997174 | 0.99721 | 21 | 17 |
| P18R1 | 0.997856 | 0.93669 | 277 | 110 |
| P18R2 | 0.968049 | 0.96555 | 81 | 65 |
| P18R3 | 0.989187 | 0.97207 | 166 | 123 |
| P18R4 | 0.998993 | 0.99814 | 26 | 6 |
| P18R5 | 0.988435 | 0.95624 | 207 | 150 |
| P1R1 | 0.991419 | 0.96089 | 221 | 98 |
| P1R2 | 0.995228 | 0.99255 | 19 | 16 |
| P1R3 | 0.99422 | 0.99255 | 21 | 11 |
| P1R4 | 0.998337 | 0.99628 | 18 | 16 |
| P20AR1 | 0.997028 | 0.97207 | 179 | 67 |
| P20AR2 | 0.993973 | 0.99162 | 31 | 24 |
| P20AR3 | 0.990421 | 0.98417 | 52 | 39 |
| P20BR1 | 0.98642 | 0.94879 | 187 | 104 |
| P20BR2 | 0.99701 | 0.93203 | 330 | 149 |
| P20BR3 | 0.986919 | 0.95717 | 123 | 92 |
| P20BR4 | 0.995423 | 0.97207 | 95 | 55 |
| P20BR5 | 0.995037 | 0.97486 | 89 | 78 |
| P21R1 | 0.993293 | 0.95345 | 227 | 77 |
| P21R2 | 0.98415 | 0.94413 | 198 | 111 |
| P21R3 | 0.999614 | 0.99441 | 24 | 13 |
| P22R1 | 0.997037 | 0.98138 | 108 | 82 |
| P22R2 | 0.994767 | 0.99441 | 16 | 9 |
| P22R3 | 0.993861 | 0.98417 | 58 | 34 |
| P22R4 | 0.997671 | 0.99069 | 48 | 29 |
| P22R5 | 0.999309 | 0.99441 | 35 | 27 |
| P22R6 | 0.998742 | 0.99441 | 24 | 17 |
| P23R1 | 0.996912 | 0.94786 | 261 | 179 |
| P24R1 | 0.996811 | 0.97858 | 112 | 36 |
| P24R2 | 0.988878 | 0.97858 | 53 | 32 |
| P24R3 | 0.997215 | 0.99534 | 13 | 9 |
| P24R4 | 0.999406 | 0.99534 | 22 | 11 |
| P24R5 | 0.998156 | 0.99814 | 26 | 13 |
| P26R1 | 0.989133 | 0.96462 | 107 | 55 |
| P26R2 | 0.991286 | 0.98324 | 55 | 38 |
| P26R3 | 0.996946 | 0.98231 | 100 | 60 |
| P26R4 | 0.989961 | 0.9851 | 48 | 39 |
| P26R5 | 0.998274 | 0.99814 | 15 | 13 |
| P27R1 | 0.99611 | 0.96462 | 159 | 120 |
| P27R2 | 0.998778 | 0.99907 | 13 | 10 |
| P29R1 | 0.995823 | 0.95345 | 232 | 100 |
| P29R2 | 0.997079 | 0.99628 | 16 | 9 |
| P29R3 | 0.998731 | 0.99534 | 22 | 13 |
| P2R1 | 0.986132 | 0.95717 | 142 | 70 |
| P2R2 | 0.996539 | 0.99534 | 19 | 10 |
| P2R3 | 0.99916 | 0.99907 | 4 | 4 |
| P2R4 | 0.998384 | 1 | 14 | 8 |
| P2R5 | 0.99162 | 0.99162 | 19 | 19 |
| P31R1 | 0.994598 | 0.97393 | 116 | 93 |
| P32AR3 | 0.995345 | 0.98976 | 55 | 25 |
| P32AR4 | 0.99948 | 0.99721 | 20 | 12 |
| P32AR5 | 0.996705 | 0.99814 | 12 | 10 |
| P36R1 | 0.996591 | 0.97207 | 203 | 83 |
| P36R2 | 0.984969 | 0.973 | 77 | 57 |
| P36R3 | 0.999674 | 0.99907 | 16 | 14 |
| P37R1 | 0.996847 | 0.97672 | 149 | 114 |
| P39R1 | 0.997184 | 0.97486 | 139 | 39 |
| P39R2 | 0.958015 | 0.94134 | 138 | 132 |
| P39R3 | 0.999719 | 0.99814 | 7 | 6 |
| P39R5 | 0.999321 | 1 | 6 | 4 |
| P3R1 | 0.996766 | 0.98603 | 55 | 21 |
| P3R2 | 0.982399 | 0.97114 | 94 | 55 |
| P3R3 | 0.995828 | 0.98138 | 111 | 41 |
| P3R4 | 0.994325 | 0.97765 | 135 | 90 |
| P3R5 | 0.996423 | 0.97114 | 139 | 93 |
| P40R1 | 0.996983 | 0.95624 | 240 | 96 |
| P40R2 | 0.995801 | 0.99255 | 23 | 19 |
| P40R5 | 0.9985 | 0.98417 | 111 | 40 |
| P40R6 | 0.996257 | 0.99534 | 21 | 19 |
| P42R1 | 0.994161 | 0.96369 | 190 | 148 |
| P42R2 | 0.995607 | 0.98696 | 61 | 32 |
| P45AR2 | 1 | 0.99721 | 23 | 23 |
| P45AR5 | 0.997446 | 0.99628 | 29 | 27 |
| P46R1 | 0.997936 | 0.9702 | 204 | 133 |
| P47R1 | 0.995641 | 0.97765 | 111 | 83 |
| P48R1 | 0.99877 | 0.99628 | 28 | 7 |
| P48R2 | 0.993684 | 0.99441 | 29 | 22 |
| P49R1 | 0.998796 | 0.95531 | 225 | 153 |
| P4R1 | 0.99407 | 0.95345 | 209 | 99 |
| P4R2 | 0.970263 | 0.96089 | 90 | 82 |
| P4R3 | 0.9968 | 0.97765 | 132 | 60 |
| P4R4 | 0.999194 | 0.99814 | 17 | 13 |
| P4R5 | 0.9991 | 0.99534 | 27 | 20 |
| P53R1 | 0.996159 | 0.97486 | 168 | 125 |
| P55R1 | 0.996826 | 0.97858 | 151 | 108 |
| P56R1 | 0.987876 | 0.973 | 112 | 84 |
| P56R2 | 0.997233 | 0.99628 | 26 | 7 |
| P56R3 | 0.997207 | 0.99534 | 11 | 9 |
| P56R4 | 0.998238 | 0.99814 | 11 | 10 |
| P5R1 | 0.988264 | 0.96276 | 110 | 60 |
| P5R2 | 0.99078 | 0.96834 | 102 | 49 |
| P5R3 | 0.988456 | 0.973 | 121 | 84 |
| P5R4 | 0.999453 | 0.99907 | 16 | 10 |
| P5R5 | 0.999707 | 0.99907 | 12 | 10 |
| P6R1 | 0.991093 | 0.973 | 117 | 56 |
| P6R2 | 0.990397 | 0.96182 | 128 | 65 |
| P6R3 | 0.998407 | 0.99255 | 35 | 18 |
| P6R4 | 0.994721 | 0.99069 | 29 | 23 |
| P7R1 | 0.997684 | 0.97393 | 193 | 127 |
| P7R2 | 0.979441 | 0.96462 | 131 | 74 |
| P7R3 | 0.988318 | 0.96369 | 109 | 49 |
| P7R4 | 0.99669 | 0.99628 | 27 | 14 |
| P7R6 | 0.998978 | 0.99534 | 8 | 7 |
| P9AR1 | 0.980543 | 0.95438 | 152 | 88 |
| P9AR2 | 0.998293 | 0.99907 | 19 | 13 |
| P9AR3 | 0.996385 | 0.99162 | 53 | 30 |
| P9AR4 | 0.985731 | 0.97579 | 109 | 81 |
| P9BR1 | 0.9734 | 0.94413 | 161 | 109 |
| P9BR2 | 0.993315 | 0.99441 | 41 | 12 |
| P9BR3 | 0.998899 | 0.99907 | 17 | 16 |
| P9BR4 | 0.997973 | 0.99721 | 15 | 9 |
| P9BR5 | 0.99815 | 0.99534 | 25 | 18 |
