# Supplementary material for: Low Microbial Diversity and Abnormal Microbial Succession Is Associated with Necrotizing Enterocolitis in Preterm Infants
Source: Front Microbiol. 2017 Nov 15;8:2243. doi: 10.3389/fmicb.2017.02243 (PMC5695202; doi:10.3389/fmicb.2017.02243)
Supplement: Supplementary file 3 [file Table_2.DOCX]

| Closest bacterial relative | Base Mean | log2 Fold Change | lfcSE | stat | p-value | OTU ID |
| --- | --- | --- | --- | --- | --- | --- |
| Firmicutes Bacilli Lactobacillales Streptococcaceae Streptococcus anginosus | 5.48 | -1.86 | 0.59 | -3.14 | 0.00 | Ten34474 |
| Bacteroidetes Bacteroidia Bacteroidales Prevotellaceae Prevotella | 2.63 | 1.46 | 0.52 | 2.81 | 0.00 | Ten15797 |
| Proteobacteria Gammaproteobacteria Pseudomonadales Pseudomonadaceae Pseudomonas | 3.63 | -1.19 | 0.60 | -1.99 | 0.05 | six29549 |

**Table S2.**
